# Synthesis, Crystal Structure, and Biological Activity of a Multidentate Calix[4]arene Ligand Doubly Functionalized by 2-Hydroxybenzeledene-Thiosemicarbazone

**DOI:** 10.3390/molecules25020370

**Published:** 2020-01-16

**Authors:** Ehsan Bahojb Noruzi, Behrouz Shaabani, Silvano Geremia, Neal Hickey, Patrizia Nitti, Hossein Samadi Kafil

**Affiliations:** 1 Department of Inorganic Chemistry, Faculty of Chemistry, University of Tabriz, 5166616471 Tabriz, Iran; ehsanbahojb@gmail.com; 2 Centre of Excellence in Biocrystallography, Department of Chemical and Pharmaceutical Sciences, University of Trieste, 34127 Trieste, Italy; nhickey@units.it (N.H.); pnitti@units.it (P.N.); 3 Drug Applied Research Center, Tabriz University of Medical Sciences, 5154853431 Tabriz, Iran; Kafilhs@tbzmed.ac.ir

**Keywords:** thiosemicarbazone, calix[4]arene, metal complex, X-ray structure, antimicrobial, anticancer

## Abstract

The design and synthesis of a novel tert-butyl-calix[4]arene functionalized at 1, 3 positions of the lower rim with two terminal 2-hydroxybenzeledene-thiosemicarbazone moieties is reported. The new ligand with multi-dentate chelating properties was fully characterized by several techniques: ESI-Mass spectroscopy, FT-IR, 1H-NMR, and single crystal X-ray diffraction. The solid state structure confirms that the calix[4]arene macrocycle has the expected open cone conformation, with two opposite phenyl rings inclined outwards with large angles. The conformation of the two alkoxythiosemicarbazone arms produces a molecule with a C2 point group symmetry. An interesting chiral helicity is observed, with the two thiosemicarbazone groups oriented in opposite directions like a two-blade propeller. A water molecule is encapsulated in the center of the two-blade propeller through multiple H-bond coordinations. The antibacterial, antifungal, anticancer, and cytotoxic activities of the calix[4]arene-thiosemicarbazone ligand and its metal derivatives (Co2+, Ni2+, Cu2+, and Zn2+) were investigated. A considerable antibacterial activity (in particular against *E. coli*, MIC, and MBC = 31.25 μg/mL) was observed for the ligand and its metal derivatives. Significant antifungal activities against yeast (*C. albicans*) were also observed for the ligand (MIC = 31.25 μg/mL and MBC = 125 μg/mL) and for its Co^2+^ derivative (MIC = 62.5 μg/mL). All compounds show cytotoxicity against the tested cancerous cells. For the Saos-2 cell line, the promising anticancer activity of ligand L (IC_50_ < 25 μg/mL) is higher than its metal derivatives. The microscopic analysis of DAPI-stained cells shows that the treated cells change in morphology, with deformation and fragmentation of the nuclei. The hemo-compatibility study demonstrated that this class of compounds are suitable candidates for further in vivo investigations.

## 1. Introduction

Calix[4]arenes are macrocyclic compounds prepared by cyclo-condensation of p-tert-butylphenols with formaldehyde under alkaline conditions [1]. The cone conformation of calix[4]arene enables them in principle to act as highly preorganized hosts, for different types of guests, leading to a vast host–guest chemistry, which can be exploited in technological applications in various fields, including the development of sensors [2], separation science [3], catalysis [4,5], and drug delivery [6,7]. The calixarene skeleton can be functionalized on both the upper and lower rims due to the presence of reactive hydroxyl groups on the lower rim and the easy substitution of tert-butyl groups on the upper rim, by reversed Friedel–Crafts reaction followed by insertion of new functional groups through electrophilic attack [8]. A wide variety of functional groups, such as carbonyl, amide, nitrile, thiourea, and Schiff-base, have been introduced onto the calixarene platform [9,10,11,12,13,14]. Functionalized calixarenes are thus able to act as hosts for neutral molecules [15], cations [16,17], and anions [18,19] and offer the possibility to act as multifunctional hosts [20,21]. For this reason, they have been widely investigated for applications based on molecular or ion recognition, including the development of highly preorganized chelating ligands for metal coordination [22,23,24,25] and hybrid materials [26,27].

The thiosemicarbazone group has been widely researched in coordination chemistry [28], analytical applications [29], and pharmacology [30]. Thiosemicarbazones usually act as flexible multi-dentate chelating ligands and can coordinate with different transition metal ions by bonding through the sulfur and hydrazinic nitrogen atoms [24,31,32,33]. Extensive investigations by different research groups have indicated that thiosemicarbazone derivatives and their transition metal complexes show remarkable biological properties, including antiviral, antimicrobial, and antitumoral activities [34,35,36,37,38,39,40,41]. Inspection of the literature shows that the functionalization of calixarenes with thiosemicarbazone has received little attention [42,43,44]. Due to the lack of calixarene-based thiosemicarbazone compounds, and considering their broad spectrum of biological applications, the design and synthesis of new thiosemicarbazone functionalized calix[4]arene compounds for use as multi-dentate chelating ligands is an area of interest. While the rigid structure of tert-butyl-calix[4]arenes means that up to four ligands can be introduced onto the framework through the hydroxy groups in a controlled manner, only two thiosemicarbazone groups would be necessary for each calixarene molecule to preorganize a coordination environment for metal ions. Therefore, in our strategy, the functionalization was limited to the 1, 3 positions of the lower rim of the tert-butyl-calix[4]arene in cone conformation, with the introduction of two terminal 2-hydroxybenzeledene-thiosemicarbazone functions, which increase the number of potential chelating atoms.

Herein, we report the synthesis, characterization, and crystal structure of a novel thiosemicarbazone calix[4]arene derivative. The biological properties, including the antimicrobial and anticancer activities of the synthesized compound and its metal derivatives (Co^2+^, Ni^2+^, Cu^2+^, and Zn^2+^), were evaluated using various microorganisms and human bone cancer cell lines (Saos-2 and MG-63). We also investigated the cytotoxic effects of the ligand and its metal derivatives on human red blood cells (HRBCs) by determination of their hemolysis rate and by application of the DAPI staining method.

## 2. Results and Discussion

### 2.1. Synthesis of the Ligand and Metal Derivatives

**Scheme 1:**
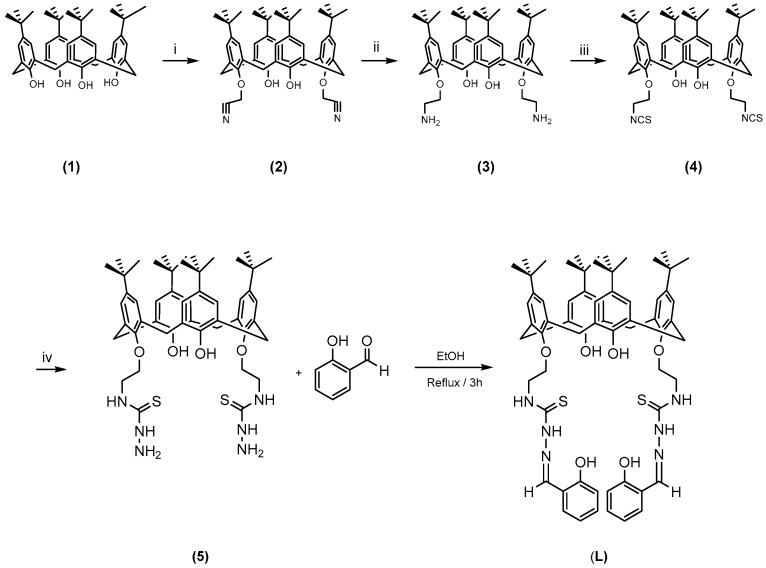
The route for synthesis of **L**. Reaction conditions: (**i**) Chloroacetonitrile, K_2_CO_3_, NaI, reflux, 7h (**ii**) LiAlH4, (0 °C), 4h (**iii**) CSCl_2_, BaCO_3_, 24h (**iv**) Hydrazine hydrate, room temperature, 3 h.

The multi-dentate thiosemicarbazone chelating ligand **L** was prepared by treatment of 5, 11, 17, 23-tetra-tert-butyl-26, 28-dihydroxy-25, and 27-bis(thiosemicarbazidoethoxy) calix[4]arene with salicylaldehyde in a 1:1 molar ratio, under reflux conditions (Scheme 1). The pale-yellow crystalline solid was obtained and further recrystallization in chloroform produced single crystals of **L** whose structure was determined by crystallography. Considering the coordinating potential of the ligand **L** toward the metal centers, several divalent transition metals (Co^2+^, Ni^2+^, Cu^2+^, Zn^2+^) were considered to form metal derivatives. To do this, we mixed one equivalent of the metal salt in MeOH with one equivalent of **L** in THF followed by reflux for 24 h. The products were finally purified by recrystallization from THF.

### 2.2. Characterization

FTIR spectra of the Ligand **L** (Figure S1) show the absorption bands corresponding to the C=N (1633 cm^−1^), N-C=S (1539 cm^−1^), and C=S (898 cm^−1^) groups [45,46]. Concerning the possible thione-thiol tautomerism in the ligand, the absence of the S-H vibration band at 2750 cm^−1^ and the presence of bands due to the C=S vibration at 898 cm^−1^ confirm that the CS group remains in the thione form [47]. The FT-IR spectra of the Co^2+^, Ni^2+^, Cu^2+^, and Zn^2+^ complexes (Figures S2–S5) were analyzed in comparison with the free ligand **L** in the region 4000–500 cm^−1^. The characteristic peaks related to the C=N and C=S groups shift to a lower wavenumber upon coordination of the metal centers to the ligand. On the other hand, the vibrations related to the N-C=S group shift to higher wavenumbers in all metal derivatives (Table S1). These changes in the C=N, N-C=S, and C=S vibrations support the suggestion of the metal coordination by **L** in all of the complexes.

**Figure 1:**
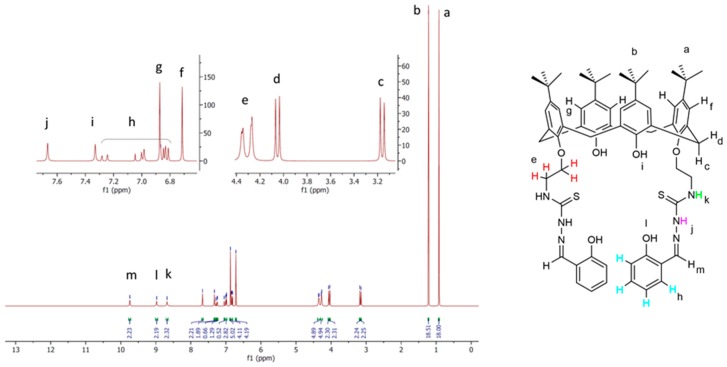
Selected portion of the ^1^H-NMR spectrum of ligand **L.**

The ^1^H-NMR spectra of ligand **L** (Figure 1) show two singlet signals for two sets of 18 t-Bu protons (δ 0.92 and 1.22 ppm), two doublet signals (δ 3.16, 4 protons, and 4.13 ppm 4 protons) assigned to the calixarene -CH_2_- bridging groups, a multiplet peak (δ 4.36–4.27 ppm) corresponding to the eight OCH_2_CH_2_NCS protons, two types of aromatic signals (δ 6.71, 4 protons, and 6.87 ppm, 4 protons) as two singlet signals assigned to the two types of calix[4]arene benzene ring protons, a multiplet peak (δ 6.83, 4 protons) and to two doublets of doublets (δ 6.99 ppm, 2 protons and 7.26 ppm, 2 protons) assigned to four types of aromatic protons of the phenol ring of the thiosemicarbazone arms, a singlet peak attributed to two OH protons of the calix[4]arene lower rim (δ 7.33 ppm), a singlet peak attributed to two thiosemicarbazone NH protons (δ 7.67 ppm), a singlet peak attributed to two NHCS protons (δ 8.68 ppm), a singlet peak attributed to two OH protons of the thiosemicarbazone phenol rings (δ 8.98 ppm), and a singlet peak related to two =CH imine protons (δ 9.74 ppm). The assignments of the ^1^H-NMR spectrum are in agreement with literature data [48].

The high-resolution mass spectra (HRMS) of ligand **L** showed molecular ion peaks corresponding to the sodium adduct of the **L** (Figure S6). The most intense peak of 1113.5315 *m/z* perfectly agrees with a compound with formula C_64_H_78_N_6_O_6_S_2_ (theoretical [L + Na]^+^ equal to 1113.5316 *m/z*) and confirms the proposed structure for **L**. HRMS spectra of the metal derivatives show in all cases the formation of the metal adduct. The Co derivative spectrum (Figure S7) is in agreement with a di-cationic complex with formula LCo(II)Co(III)^2+^, formed by the loss of 3 H^+^ from L, [C_64_H_75_N_6_O_6_S_2_Co_2_]^2+^. Analogous partial oxidation of the Co^2+^ species was also observed for the compound **5** derivative [49]. The HRMS spectrum of the Ni derivative (Figure S8) reveals the presence of multiple species, including a mixture of monomeric and dimeric complexes of a mono-deprotonated ligand, with formula LNi(II)^+^ and L_2_Ni(II)_2_^2+^, [C_64_H_77_N_6_O_6_S_2_Ni]^+^ and [C_128_H_154_N_12_O_12_S_4_Ni_2_]^2+^ (Figure S9), and a di-cationic complex of L^2−^ with formula LNi(II)_2_^2^, [C_64_H_76_N_6_O_6_S_2_Ni_2_]^2+^. The HRMS spectrum of the Cu derivative (Figure S10) shows the highest peak, which is consistent with an analogue di-cationic specie of the doubly deprotonated L ligand of formula LCu(II)_2_^2+^, [C_64_H_76_N_6_O_6_S_2_Cu_2_]^2+^. Finally, the highest peak of the HRMS spectrum of the Zn derivative (Figure S11) is also attributable to a di-cationic species with the formula LZn(II)_2_^2+^, [C_64_H_76_N_6_O_6_S_2_Zn_2_]^2+^.

The elemental analysis are consistent with nitrate salts of di-cationic species of the general formula [LM](NO_3_)_2_, with the exception of the Co derivative. In this case, the elemental analysis data can be rationalized as a di-hydrated nitrate salt [LCo](NO_3_)_2._ 2H_2_O (Table S3).

### 2.3. Solid-State Structure of L

The X-ray structure of **L**, determined using synchrotron radiation with cryogenic techniques, shows that the asymmetric unit of the centrosymmetric triclinic crystals is composed of one molecule of **L**, a water molecule bound by multiple H-bond interactions, a co-crystallized chloroform solvent molecule disordered over at least two positions, and a partially occupied co-crystallized chloroform solvent molecule in close proximity to a crystallographic inversion center. Crystallographic data and refinement details are reported in Table S2.

**Figure 2:**
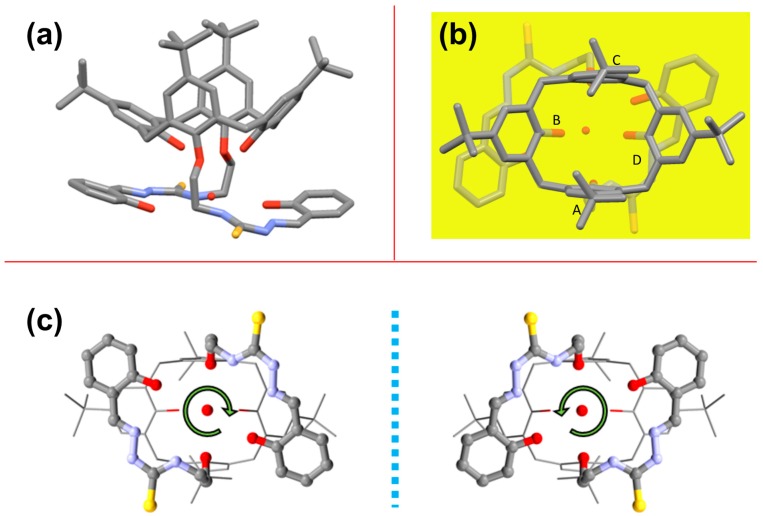
Solid-state structure of **L**. (**a**) The molecule shows a cone conformation. (**b**) Orthogonal view of **L** with respect to the calix[4]arene mean plane (yellow) defined by the methylene bridging groups. (**c**) The two enantiomeric conformations of **L** observed in the centrosymmetric crystal structure. Hydrogen atoms have been omitted for clarity.

The calix[4]arene macrocycle shows the expected cone conformation (Figure 2a). The mean planes of the two phenyl rings with alkoxythiosemicarbazone substituents (**A** and **C**) are almost orthogonal to the calix[4]arene mean plane, defined by the methylene bridging groups, with slight outward dihedral angles of 96° and 97°, respectively, (Figure 2b). With regard to the hydroxy-substituted phenyl rings (**B** and **D**), the mean planes of both phenyl rings are also both inclined outwards with respect to the calix[4]arene mean plane, with larger dihedral angles of 137° and 147°, respectively. Consequently, the cone exhibits a quite open overall conformation. The similar values of the above-discussed pairs of dihedral angles reflect the C2 symmetry of the calix[4]arene derivative symmetrically functionalized in the 1, 3 positions of the lower rim. This open structure is largely due to the formation of two intramolecular H-bonds between the hydroxy groups as donors and the adjacent alkoxy oxygen atoms as acceptors, with O∙∙∙O distances of 2.871 (ring B-ring A) and 2.922 Å (ring D-ring C) (Table 1). Thus, the substitution pattern on the lower rim strongly influences the conformation of the calix[4]arene cone.

The conformations of the two alkoxythiosemicarbazone substituents on rings B and D are very similar, with comparable values of corresponding torsion angles along the chains. More specifically, the entire conformation of the arms is largely determined by the positive (or negative in the other enantiomeric conformer present in the centrosymmetric crystal structure) gauche conformation of both the ethyl spacer (61.4 and 57.6° for A and C, respectively). As a result, the thiosemicarbazone groups are oriented in opposite directions like a two-blade propeller, reflecting the C2 point group symmetry of the molecule, thereby forming an interesting chiral helicity (Figure 2c).

Overall, the entire thiosemicarbazone substituents, including their terminal phenol groups, tend towards coplanarity: The mean planes of the two NCNN groups of atoms exhibit a dihedral angle of 19° while the dihedral angles between the mean plane of the NCNN atoms and the mean plane of its terminal phenyl ring are 19° and 13° for **A** and **C**, respectively. Similarly to the situation regarding the conformation of the cone, this coplanar conformation is influenced by the formation of two intramolecular H-bonds between the hydroxy groups of the terminal phenol groups as donors and the imine N atom of the thiosemicarbazone as an acceptor, with O∙∙∙N distances of 2.710 (ring A substituent) and 2.742 Å (ring C substituent) (Table 1).

A significant amount of electron density approximately at the center of the oppositely oriented thiosemicarbazone groups was interpreted and well modelled as a water molecule. The water oxygen atom is within the H-bond distance of a number of the potential chelating atoms at various positions on the lower rim, including the N atoms of the thiosemicarbazone and hydroxy oxygens on both the terminal phenol groups and the calix[4]arene lower rim, as indicated in Table 1, in which details of the main short interactions observed in the crystal structure are summarized. This central water molecule, mimicking a possible coordinated metal ion, is surrounded by eight N,O atoms with the formation of multiple H-bonds, although some are very weak. It should be noted that water was not used as a solvent so its presence is presumably explained as an impurity of the methanol solvent or as adsorption from the atmospheric humidity.

Finally, the crystal packing shows one intermolecular H-bond between the hydroxy of one of the thiosemicabazone terminal phenol groups (ring **C**) and its inversion-related hydroxy group, with an O∙∙∙O distance of 2.927 Å.

The overall conformation of L and the easy inclusion of the water molecules indicates the potential of **L** as a polydentate chelating ligand.

### 2.4. Determination of Antimicrobial and Antifungal Activities

All of the synthesized compounds (**L** and Co, Ni, Cu, and Zn complexes) were evaluated for their quantitative antibacterial activity in the serial double dilution method against gram-positive bacteria (*S. aureus* and *B. subtilis*) and gram-negative bacteria (*E. coli* and *P. aeruginosa*). The anti-fungal activity was also investigated (*C. albicans* and *C. glabrata*). Gentamicin and nystatin were used as standard antibacterial and antifungal drugs, respectively. Considering literature data [50] and according to Table 2, all of the derivatives showed strong antibacterial activity against the tested bacteria except *S. aureus,* which only showed sensitivity against the copper complex. In many cases, the MIC of the synthesized compounds coincides with the MBC (31.25 μg/mL), which confirms the bactericidal antimicrobial activity of the ligand and its related coordination compounds. In general, the fungal strains were resistant against the synthesized compounds except for ligand L and its cobalt complex, which respectively showed strong and mild antifungal activity against *C. albicans.*

Comparing these results with our recently published paper [49] reveals that the thiosemicarbazone schiff-base ligand is a stronger antimicrobial agent than its thiosemicarbazide analogue. Its antibacterial activity (MIC value) increased 8-fold and 2-fold against *E. coli* and *P. aeruginosa*, respectively, in comparison with thiosemicarbazide. It also showed a significant antifungal activity against *C. albicans* while this fungal strain was resistant against the thiosemicarbaide ligand. In the case of metal derivatives, generally all of the thiosemicarbazone derivatives are stronger antibacterial agents than their corresponding thiosemicarbazide derivatives. The antibacterial activity of the copper derivative is eight times stronger than the thiosemicarbazide analogue against *S. aureus* and the cobalt derivative showed strong antimicrobial activity against *B. subtilis* strain while this bacteria was resistant against the corresponding thiosemicarbazide derivative. Ferthermore, the antimicrobial activity of the zinc derivative increased four-fold in comparison with its thiosemicarbazide derivative. In the case of gram-negative bacteria (*E. coli* and *P. aeruginosa*), the improvement of the antibacterial activities of the metal derivatives are more pronounced in comparison to the corresponding thiosemicarbazide derivatives. The MIC values of the Co, Ni, and Cu derivatives are modified 4 times, 2 times, and 4 times against *E. coli* bacteria, respectively, in comparison with their thiosemicarbazide equivalents. Considering *P. aeruginosa*, the MIC values of the Co and Cu derivatives are improved 4 times and 2 times, respectively.

### 2.5. Cytotoxicity Assay

**Figure 3:**
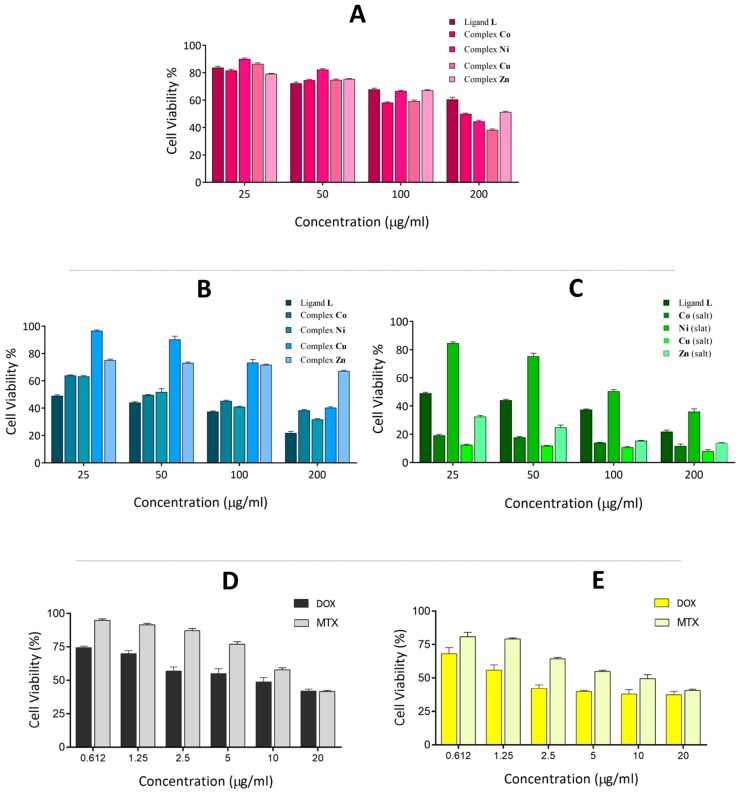
Cytotoxicity evaluation of synthesized compounds at various concentrations against cancer cell lines; (**A**) (MG-63 cell lines), (**B**), and (**C**) (Saos-2 cell lines). Cytotoxicity evaluation of MTX and DOX at various concentrations against MG-63 (**D**) and Saos-2 (**E**) cell lines.

To determine the in vitro cytotoxicity, the IC_50_ values of the calix[4]arene-based ligand (**L**) and its complexes were evaluated against two different human bone cancer cell lines (Saos-2 and MG-63) by the MTT reduction assay. All of the compounds were dispersed in water (and 10% DMSO) and diluted with cell culture medium to reach four required concentrations of 25, 50, 100, and 200 μg/mL. The viability of cancer cells versus the compound concentrations is shown in Figure 3. In general, a significant dose-dependent antiproliferative activity was observed with the exclusion of the Zn derivative against Saos-2 cells. Considering the Saos-2 cell line, the anticancer activity of ligand **L** is higher than its metal derivatives at all concentrations, especially at a higher dosage (about 21%). With the exception of the Zn complex, all of the compounds showed increasing cytotoxicity against cancerous cells with an increasing concentration. 

The copper derivate is nearly non-toxic at lower concentrations (around 96%), but its toxicity increased to a level comparable with the Ni and Co derivatives (nearly 39% at 200 μg/mL). In the case of the MG-63 cell line, all of the compounds showed very low toxicity at a lower dosage (80% on average), but their toxicity considerably changed with increasing concentration, especially the copper derivate, which showed 38% cell viability at the concentration of 200 μg/mL. In general, the anticancer activity of thiosemicarbazone compounds could be the consequence of the presence of the imine group in their structure [51] and to the intercalation between pairs of DNA bases, or the breaking of DNA strands [52,53].

Methotrexate (MTX) and doxorubicin (DOX) are well-known anti-cancer drugs, which are routinely used by research groups as effective anti-cancer drugs against human bone tissue cancer cell lines [54,55,56,57]. Herein, we used both of these anti-cancer drugs (with serial dilution from 20 to 0.612 μg/mL) as a well-studied criterion to compare our synthesized compound’s cytotoxicity with their anti-cancer activities. As shown in Figure 3D,E, both of these drugs show strong anti-cancer effects against Saos-2 and MG-63 cell lines, especially at the concentration range 2.5–20 μg/mL. Although all of our compounds are not as strong anti-cancer agents as these drugs, the free ligand **L** and its Co, Ni, and Cu derivatives could be considered as somewhat effective anti-cancer agents against the Saos-2 cancer cell line with relatively low IC_50_ (Table 3).

The IC_50_ values were calculated from the curves (dose–response curves) constructed by plotting the cell viability (%) versus the concentration (μg/mL); therefore, the IC_50_ is equal to the concentration at which the cell viability equals 50%. The results of the cytotoxic activity (IC_50_ (μg/mL)) are summarized in Table 3. Ligand **L** is more toxic against Saos-2 cells with a lower IC_50_ value (<25 μg/mL) and the copper derivate is more toxic against the MG-63 cell with IC_50_ = 140 μg/mL.

Control assays performed with the corresponding inorganic salts show that the activity is mainly ascribable to the inorganic component rather than the organic calixarene component. Actually, the **L** ligand appears to protect the cells against the inherent cytotoxicity of the bivalent ions present in the inorganic salts of these metals.

Comparison of these results with those obtained from our previously published thiosemicarbazide ligand and its metal derivatives [49] indicates that the thiosemicarbazone Schiff-based ligand is a more effective anti-cancer agent against Saos-2 cancer cells versus thiosemicarbazide ligand (IC_50_ < 25 μg/mL versus IC_50_ > 200 μg/mL) even at lower concentrations. Considering the present Ni and Cu derivatives, it is apparent that the IC_50_ values (as a criterion of anticancer activity) decreased 3.22-fold (decrease from 200 μg/ml to 62 μg/ml) and 3.95-fold (decrease from 170 μg/mL to 43 μg/mL), respectively, in comparison with their thiosemicarbazide analogues.

### 2.6. Morphology of Cell Nuclei

**Figure 4:**
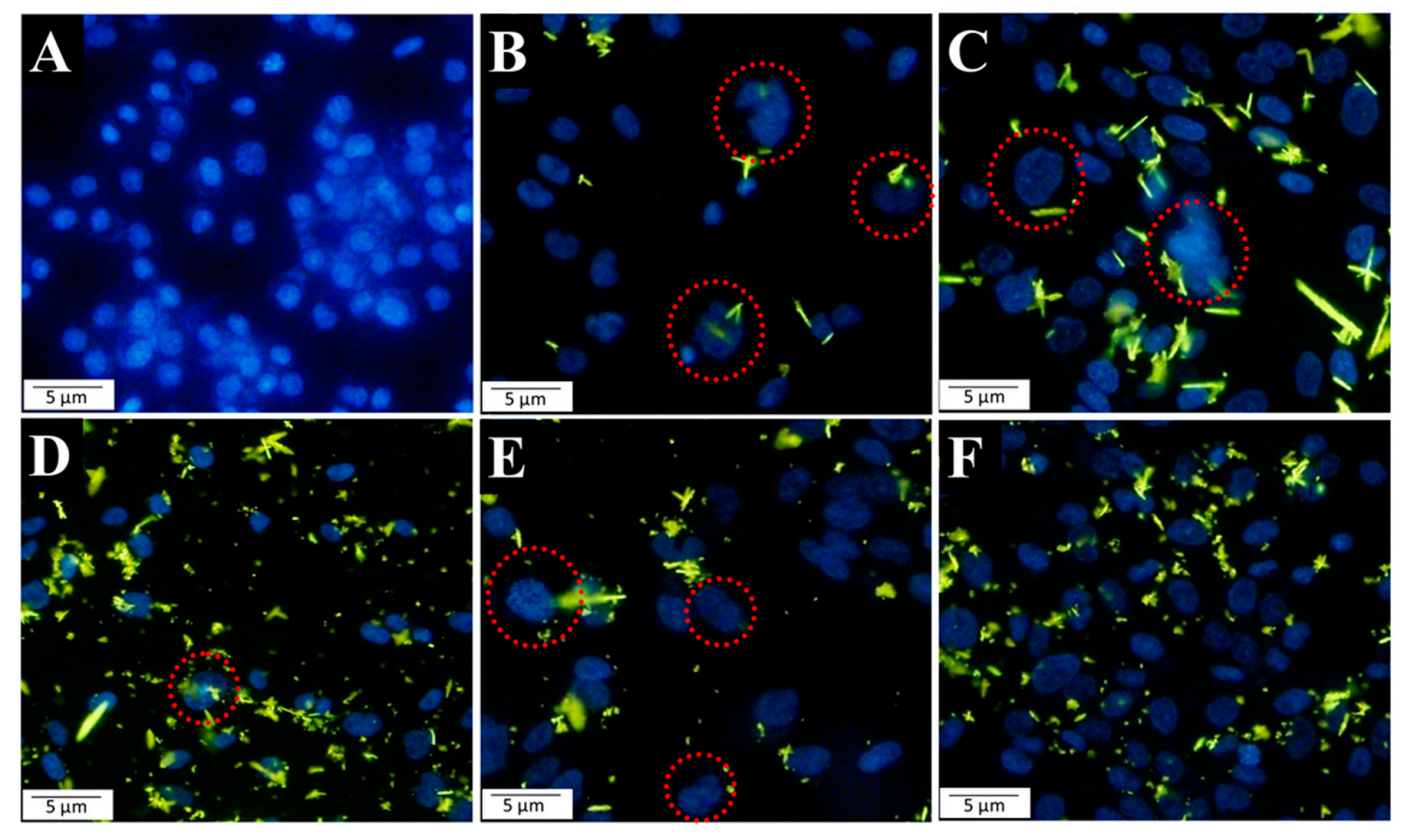
Fluorescent microscopic images of DAPI-stained cells following 48-h treatment with **A** (fresh medium (control)), **B** (ligand **L**), **C** (complex Co^2+^), **D** (complex Ni^2+^), **E** (complex Cu^2+^), and **F** (complex Zn^2+^). Red circles indicate the Saos-2 nuclei with anomalous morphology after internalization of the fluorescent chemical compounds (**L**, and complexes of Co^2+^, Ni^2+^, Cu^2+^, and Zn^2+^).

The morphology, shape, and condensation of chromatin is a good indicator of healthy apoptotic and necrotic cells [6]. According to MTT assay data, our synthesized compounds have considerable anticancer activity against Saos-2 cancer cells. With this in mind, the apoptosis induction properties of the samples upon treatment of the Saos-2 bone cancer cell line were investigated by microscopic analysis of DAPI-stained cells. Microscopic images of DAPI-stained cells following 48 h exposure to control media (**A**), ligand **L** (**B**), complex Co^2+^ (**C**), complex Ni^2+^ (**D**), complex Cu^2+^ (**E**), and complex Zn^2+^ (**F**) are shown in Figure 4.

The untreated control Saos-2 cells showed normal nuclei and no necrosis of cancer cells nucleus was observed. Whereas **L** and complex (Co^2+^, Ni^2+^, and Cu^2+^)-treated cells showed deformed and fragmented nuclei in their chromatin and consequently, the morphology of the cells was changed. On the other hand, the cancer cells treated with complex Zn^2+^ retained their normal structures, and their morphology and the number of cells were unchanged. Considering the DAPI images of cells treated with **L** and Cu^2+^ derivate, it is evident that the number and the shape of cancer cells decreased and changed drastically, respectively, which is in good agreement with their IC_50_ and higher anticancer activity.

### 2.7. Biocompatibility Evaluation

**Figure 5:**
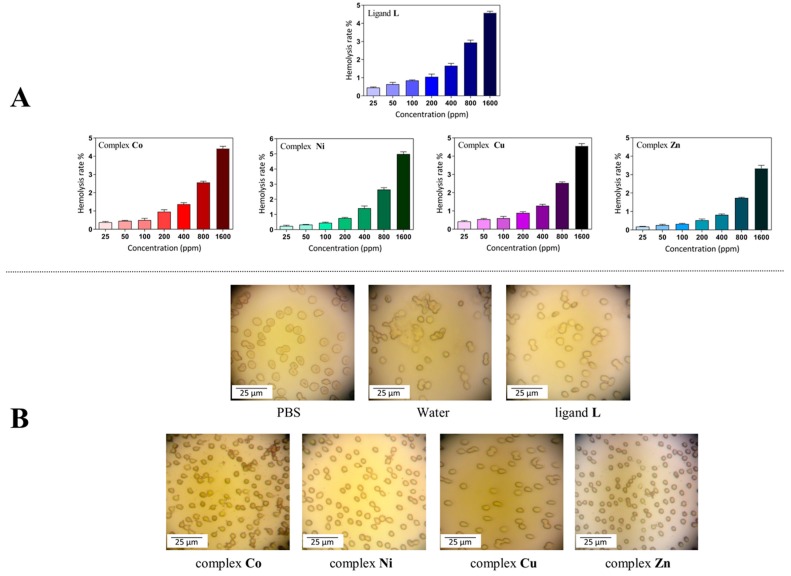
Hemolysis rate of HRBCs in the presence of synthesized compounds at different concentrations (**A**). Optical images of HRBCs treated with synthesized compounds (at the concentration of 200 μg/mL) under light microscopy (**B**).

In biomedical applications, the effect of synthesized chemical compounds (drugs, drug vehicles, nano-materials) on live tissues and cells (blood cells) is a critical parameter. Different experiments, including the hemolysis assay, coagulation, erythrocytes aggregation, and sedimentation rate, can be used to evaluate the biocompatibility of synthesized materials. In this regard, the hemocompatibility of the **L** and its metal derivatives was investigated by measuring hemolytic activity. To do this, the hemolytic effects of prepared compounds evaluated at the various concentrations in the range 25–1600 μg/mL on HRBCs at physiological conditions (pH 7.4 and 37 °C) and the results are shown in Figure 5. As is clear in Figure 5A, dose-dependent hemolytic effects of all chemical compounds on HRBCs were observed, and the results confirmed that there are only slight hemolytic effects from the synthesized materials even at higher concentrations. For further inspection of the impact of the synthesized compounds on HRBCs, the blood samples were treated with the compounds under investigation at a moderate concentration (200 μg/mL), PBS, and deionized water. The optical microscopy images revealed that the HRBCs shapes show no significant changes after treatment with **L** and its metal complexes for 3 h and they retain their disc-like structure with almost intact erythrocytes membranes. The HRBCs treated with PBS (negative control) and water (positive control) showed diskocytes and lysis forms, respectively (Figure 5B). In general, the results of the hemo-compatibility study confirmed that our synthesized compounds have a very slight lysis effect on HRBCs at lower concentrations and also their hemolytic effects is lower than the permissible range (<4.5%) [58] even at a higher dosage, which would make them suitable candidates for further in vivo investigations.

## 3. Materials and Methods

### 3.1. Reagents

All chemical reagents and solvents were purchased from Merck/Aldrich and were used without further purification. Human blood was obtained from the Iranian Blood Transfusion Institute. Mueller Hinton Agar (MHA) and Mueller Hinton Broth (MHB) were purchased from Quelab and Merck, respectively. Roswell Park Memorial Institute 1,640 growth medium (RPMI) was purchased from Gibco BRL Life Technologies. Doxorubicin (DOX) was obtained from Sobhan Pharmaceuticals Co. (Tehran, Iran). Methotrexate (MTX), gentamicin, and nystatin were purchased from Zahravi Pharmaceuticals Co. (Tabriz, Iran). All microorganisms strains, namely, *Staphylococcus aureus* (ATCC^®^ 29213™), *Bacillus subtilis* (ATCC^®^ 6633™), *Escherichia coli* (ATCC^®^ 25922™), *Pseudomonas aeruginosa* (ATCC^®^ 27853™), *Candida albicans* (ATCC^®^ 10231™), and *Candida glabrata* (ATCC^®^ 2001™), were provided from Persian Type Culture Collection (PTCC, Karaj, Iran), and the microbiology department of the Drug Applied Research Centre (Tabriz, Iran).

### 3.2. Instrumentation

Fourier-transform infrared (FT-IR) spectra were recorded with a Bruker Tensor 27 spectrometer (Karlsruhe, Germany) in the region 4000–500 cm^−1^ using KBr pellets. Proton nuclear magnetic resonance (^1^H-NMR) spectra were recorded in CDCl_3_ on a Bruker Spectrospin Avance 400 MHz ultra-shield spectrometer (Karlsruhe, Germany) and Varian 400 MHz (Palo Alto, CA, USA). Mass spectra were recorded on an ion trap instrument Bruker esquire 4000 (Karlsruhe, Germany). An Olympus Bh2-RFCA fluorescence microscope (Tokyo, Japan) was used to evaluate the results of the DAPI staining procedures (vide infra). Microanalyses were carried out using a Heraeus CHN–O–Rapid analyzer (Hanau, Germany). Melting points were measured on an Electrothermal 9100 apparatus (Stone, UK). Crystallographic data were collected on the XRD1 diffraction beam-line of the ELETTRA Synchrotron (Trieste, Italy).

### 3.3. Synthesis

#### 3.3.1. Preparation of Ligand

Literature procedures for the synthesis of *p*-tert-butylcalix[4]arene (1), 1,3 dinitrile-substituted *p*-tert-butylcalix[4]arene (2), 1,3 diamine-substituted *p*-tert-butylcalix[4]arene (3), and 1,3 diisocyanate-substituted *p*-tert-butylcalix[4]arene (4) were followed [48,59,60]. Bisthiosemicarbazide-substituted *p*-tert-butylcalix[4]arene (**5**) was prepared as described in our recent paper [49].

Synthesis of 5, 11, 17, 23-tetra-tert-butyl-26, 28-dihydroxy-25, 27-bis(2(2-hydroxybenzeledene)thiosemicarbazonoethoxy) calix[4]arene (**L**)

A solution of salicylaldehyde (0.48 mL, 44 mmol) in ethanol (5 mL) was added to a solution of compound **5** (0.50 g, 5.60 mmol) in ethanol (25 mL). A yellow powder was formed. The mixture was refluxed for 6 h, after which the solution cooled to room temperature and the precipitate was filtered and washed with cold ethanol to remove unreacted aldehyde. Yield 78%. mp: 230 °C (dec.). FTIR (KBr, cm^−1^); 468, 570, 681, 744, 897, 968, 1035, 1153, 1200, 1266, 1318, 1372, 1479, 1538.7, 1632, 1694, 1944, 2633, 2743, 2955, 3159, 3412, ESI-HRMS: LNa^+^, [C_64_H_78_N_6_O_6_S_2_+Na]^+^ calcd: 1113.5316 *m/z*; found:1113.5315 *m/z*; ^1^H-NMR: (400 MHz, CDCl_3_, TMS, 25 °C, δ ppm), 9.74 (s, 2 H; =CH-), 8.98 (s, 2 H; OH), 8.68 (s, 2 H; -NHCS), 7.67 (s, 2 H; OH), 7.33 (s, 2 H; -CSNH), 7.26 (m, 2H, aldehyde ring), 6.99 (d, 2H, aldehyde ring), 6.87 (s, 4 H; ArH), 6.83 (m, 4H, aldehyde ring), 6.71 (s, 4 H; ArH), 4.13 (d, 4 H; ArCH_2_Ar), 4.36–4.27 (m, 8 H; OCH_2_CH_2_NCS), 3.16 (d, 4 H; ArCH_2_Ar), 1.22 (s, 18H; t-Bu), 0.92 (s, 18H; t-Bu). Anal. Calcd. for C_64_H_78_N_6_O_6_S_2_ (1091.48): C, 70.43; H, 7.20; N, 7.70; Found: C, 70.13; H, 7.08; N, 7.57.

#### 3.3.2. Preparation of Metal Derivatives

All of the metal derivatives were synthesized according to the following procedure: A solution of metal salt containing Co(NO_3_)_2_.6H_2_O (0.026 g), Ni(NO_3_)_2_.6H_2_O (0.026 g), Cu(NO_3_)_2_.3H_2_O (0.022 g) or Zn(NO_3_)_2_.4H_2_O (0.024 g) in methanol (5 mL) and ligand L (0.1 g) in THF (10 mL) was stirred and refluxed for 24 h. The precipitate was then filtered and the solvent of the filtrate was evaporated under reduced pressure. Finally, the obtained solid was purified by crystallization using THF.

##### Cobalt Derivative: 

Brown, Yield: 84%. mp: 244 °C (dec.). FTIR (KBr, cm^−1^), 588, 635, 674, 782, 819, 873, 920, 1039, 1120, 1199, 1384, 1482, 1637, 2871, 2958, 3395. ESI-HRMS: LCo(II)Co(III)^2+^, [C_64_H_75_N_6_O_6_S_2_Co_2_]^2+^. calcd: 602.6921 *m/z*; found: 602.6922 *m/z*. Anal. Calcd. for [CoL](NO_3_)_2_.2H_2_O C_64_H_82_N_8_O_14_S_2_Co (1310.44): C, 58.66; H, 6.31; N, 8.55; Found: C, 58.76; H, 5.97; N, 8.43.

##### Nickel Derivative: 

Orange, Yield: 68%. mp: 214 °C (dec.). FTIR (KBr, cm^−1^), 587, 685, 747, 817, 873, 922, 1038, 1121, 1197, 1238, 1370, 1481, 1563, 1634, 2095, 2957, 3278. ESI-HRMS: LNi(II)_2_^2+^, [C_64_H_76_N_6_O_6_S_2_Ni_2_]^2+^ calcd: 603.1972 *m/z*; found: 603.1950 *m/z*. Anal. Calcd. for [NiL](NO_3_)_2_ C_64_H_78_N_8_O_12_S_2_Ni (1274.17): C, 60.33; H, 6.17; N, 8.79; Found: C, 60.32; H, 5.95; N, 8.99.

##### Copper Derivative: 

Green-brown, Yield: 53%. mp: 210 °C (dec.). FTIR (KBr, cm^−1^), 583, 632, 689, 806, 873, 1034, 1108, 1196, 1303, 1367, 1479, 1576, 1635, 2957, 3046, 3397. ESI-HRMS: LCu(II)_2_^2+^, [C_64_H_76_N_6_O_6_S_2_Cu_2_]^2+^ calcd: 608.1923 *m/z*; found: 608.1903 *m/z*. Anal. Calcd. for [CuL](NO_3_)_2_ C_64_H_78_N_8_O_12_S_2_Cu (1279.02): C, 60.10; H, 6.15; N, 8.76; Found: C, 59.72; H, 5.98; N, 8.66.

##### Zinc Derivative: 

Brown, Yield: 91%. mp: 208 °C (dec.). FTIR (KBr, cm^−1^), 581, 674, 783, 818, 873, 1036, 1115, 1198, 1373, 1481, 1635, 2564, 2958, 3270. ESI-HRMS: for LZn(II)_2_^2+^, [C_64_H_76_N_6_O_6_S_2_Zn_2_]^2+^ calcd: 610.1905 *m/z*; found: 610.1903 *m/z*. Anal. Calcd. for [CuL](NO_3_)_2_ C_64_H_78_N_8_O_12_S_2_Zn (1280.87): C, 60.01; H, 6.14; N, 8.75; Found: C, 60.23; H, 6.01; N, 8.64.

### 3.4. Crystal Structure Determination and Refinement of **L**

Small single crystal rods were obtained by slow evaporation of a chloroform solution containing **L**. Data collection was carried out at the Macromolecular crystallography XRD1 beamline of the Elettra synchrotron (Trieste, Italy), employing the rotating-crystal method with a Dectris Pilatus 2M area detector. Single crystals were dipped in PEG200 cryoprotectant, mounted on a loop, and immediately flash-frozen under a liquid nitrogen stream at 100 K. The use of synchrotron radiation with crystals cooled to 100 K was necessary to obtain a crystal structure at atomic resolution. The pure diffraction is due to the small crystal size (rods with typical dimensions of 0.1 × 0.05 × 0.02 mm), as well as the significant solvent disorder observed (see below). Diffraction data were indexed and integrated using the XDS package [61] while scaling was carried out with XSCALE [49]. The structure was solved using the SHELXT program [62] and structure refinement was performed with SHELXL-14 by full-matrix least-squares (FMLS) methods on F^2^ [63] operating through the WinGX GUI [64].

The crystal structure shows the presence of highly disordered co-crystallized solvent chloroform molecules in voids created by the crystal packing. In particular, in the asymmetric unit, a co-crystallized chloroform solvent molecule disordered over at least 2 positions and a partially occupied co-crystallized chloroform solvent molecule in close proximity to a crystallographic inversion center were found. The PLATON SQUEEZE procedure was used to remove the electron density related to these highly disordered chloroform molecules [65]. The residual electron density of 169 electrons/cell in a total potential solvent area volume of 652.7 (19.1% of the cell volume) can be attributed to 2.9 chloroform solvent molecules (1.45 molecules in the asymmetric unit of the centrosymmetric triclinic crystal).

All non-hydrogen atoms were refined anisotropically. The hydrogen atoms of the water molecule were refined using DFIX and DANG instructions to restrain the OH bond lengths and HOH angle while all other hydrogen atoms were added at the calculated positions and refined using the riding model.

Crystal Data for C64H78N6O6S2·H2O (M = 1109.46 g/mol): triclinic, space group P-1 (no. 2), a = 12.690(6) Å, b = 15.344(18) Å, c = 19.15(3) Å, α = 104.44(4)°, β = 98.01(4)°, γ = 104.016(10)°, V = 3423(6) Å^3^, Z = 2, T = 100(2) K, μ = 0.122 mm^−1^, D_calc_ = 1.076 g/cm^3^, 23853 reflections measured, 6918 unique (R_int_ = 0.1328) which were used in all calculations. The final R_1_ was 0.0683 (I > 2σ(I)) and wR_2_ was 0.2108 (all data).

CCDC 1944727 contains supplementary crystallographic data for this paper. These data can be obtained free of charge via http://www.ccdc.cam.ac.uk/conts/retrieving.html (or from the CCDC, 12 Union Road, Cambridge CB2 1EZ, UK; Fax: +44-1223-336033; E-mail: deposit^@^ccdc.cam.ac.uk)

### 3.5. Antimicrobial Studies

#### 3.5.1. Preparation of Microorganism’s Suspensions 

*S. aureus* (ATCC^®^ 29213™), *B. subtilis* (ATCC^®^ 6633™), *E. coli* (ATCC^®^ 25922™), *P. aeruginosa* (ATCC^®^ 27853™), *C. albicans* (ATCC^®^ 10231™), and *C. glabrata* (ATCC^®^ 2001™) were cultured onto sterile Mueller-Hinton agar plates, and incubated at 37 °C for 24 h. After incubation, a single colony was selected to inoculate a sterile tube containing 2 mL of sterile normal saline to match the turbidity of a 0.5 McFarland standard (10^8^ cfu/mL). Further dilution was done with sterile normal saline to 10^5^ cfu/mL before incubation.

#### 3.5.2. Broth Microdilution Method

Serial dilution of the synthesized compounds suspension in DMSO (2000–15.62 μg/mL) was done in a sterile 96-well plate. Then, 20 μL of an overnight bacterial and fungal suspension and 180 μL of the compounds suspension in MHB (and 1% glucose for fungal strains) were mixed in each well. The final concentration of DMSO was 10%. After 24 h of incubation at 37 °C, the first concentration with no bacterial growth is regarded as the minimum inhibitory concentration (MIC). Controls (microorganism strain in MHB without chemical and MHB alone) were included in each measurement. Aliquots of 5 μL from wells in which no growth was observed were spotted onto Mueller–Hinton agar plates to determine the minimum bactericidal concentration (MBC) values. The MBC was read as the lowest concentration with no growth after 24 h of incubation [66].

### 3.6. Cell Culture and MTT Assay

The colorimetric MTT assay, introduced by Mossmann [67], was used for the evaluation of the anti-cancer activity of our synthesized compounds against Saos-2 and MG-63 cell lines (human bone cancer cells). These cells were obtained from the Pasteur Institute of Iran, Tehran, Iran and maintained in RPMI 1640 medium supplemented with 10% FBS and 1% benzylpenicillin/streptomycin. Further, the Saos-2 and MG-63 cell lines were seeded at a density of 1 × 10^4^ cells/well in 96-well plates and maintained at 5% CO_2_ in a CO_2_ incubator at 37 °C for 24 h, after which the medium was replaced with fresh medium containing different concentrations (200, 100, 50, and 25 μg/mL) of Ligand L and complexes (Co, Ni, Cu, and Zn). The cells without treatment were considered as the control. After 48 h, the medium was removed and replaced with 200 μL of fresh medium containing 100 μg mL^−1^ MTT powder and incubated for a further 4 h. The medium was then withdrawn and 100 μL of DMSO was added to dissolve the formazan crystals inside the cells. The absorbance of solubilized formazan was detected at 570 nm with the reference wavelength of 630 nm using an ELISA plate reader (Stat Fax, 2100, Awareness Technology Inc., Palm City, FL, USA) [68]. The viability of cells in each well was calculated by the following formula:

(1) $$\mathrm{viability}\;\mathrm{of}\;\mathrm{cells}\;\left(\%\right)=\frac{\mathrm{mean}\;\mathrm{absorbance}\;\mathrm{of}\;\mathrm{sample}}{\mathrm{mean}\;\mathrm{absorbance}\;\mathrm{of}\;\mathrm{control}}\times 100.$$

### 3.7. DAPI Staining

To visually study the apoptotic effects of the synthesized compounds (L and its Co, Ni, Cu, and Zn complexes), the 4′,6-diamidino-2-phenylindole (DAPI) staining assay was performed according to the literature procedure [58]. Briefly, Saos-2 cells were seeded in six-well plates (5 × 10^4^ cells per well) and incubated at 37 °C for 24 h. After the growth of the cells, the culture media were replaced by fresh media containing L and its metal derivatives (sterile filtered through 500 nm) at their half-maximal inhibitory growth concentration (IC_50_). Cells receiving no treatment were considered as the control group. After 48 h, the cells were washed three times with fresh PBS (pH 7.4) to remove any chemical compounds. Subsequently, the cells were fixed with 4 wt% paraformaldehyde for 15 min at room temperature. Afterwards, the cells were washed three times with PBS and permeabilized with Triton X-100 (0.1% *w/v*) for 5 min. The cells were washed again with PBS and stained with 300 ng mL^−1^ DAPI for 15 min. DNA condensation and fragmentation in apoptotic cells were evaluated under a fluorescence microscope.

### 3.8. Erythrocytes Hemolysis Assay

Ligand **L** and all of the complexes at different concentrations were subjected to a hemolysis assay to assess the toxicities of synthesized compounds to human red blood cells (HRBCs). The erythrocytes hemolysis assay was conducted according to the method of Shafiei et al. [69]. In this regard, fresh human blood was received from IBTI and centrifuged at room temperature (4000 rpm, 10 min). The obtained erythrocytes were washed three times with 10 mL of phosphate-buffered saline (PBS) to remove blood proteins and serum from the RBCs. The total isolated RBCs were diluted 10 times with PBS. In each microtube, 0.5 mL of ligand **L** and all of the complexes at different concentrations (25, 50, 100, 200, 400, 800, 1600 μg/mL) and 0.5 mL of diluted RBCs were mixed and incubated at 37 °C for 3 h in an incubator shaker. Hemolysis was measured by analyzing the absorbance of free hemoglobin leaked out of compromised RBCs in the supernatants at 540 nm. The RBCs incubated with PBS and water (1:1) were used as 0% and 100% hemolysis controls. The hemolysis rate was calculated via the following formula:

(2) $$\mathrm{Hemolysis}\left(\%\right)=\frac{A_{\mathrm{Sample}}-A_{\mathrm{PBS}}}{A_{\mathrm{Water}}-A_{\mathrm{PBS}}}\times 100.$$

## 4. Conclusions

In this study, a novel calix[4]arene-based thiosemicarbazone derivative with multi-dentate chelating properties was synthesized and characterized by NMR, single crystal X-ray structure analysis, and HRMS. Both the 1H-NMR spectra and solid state structure confirmed that the calix[4]arene macrocycle has the cone conformation. In particular, the X-ray structure shows that the cone exhibits a typical open conformation, with two opposite phenyl rings inclined outwards with large angles. The similar gauche conformation of the two ethyl spacers of the alkoxythiosemicarbazone substituents results in a C2 point group symmetry molecule, with the two thiosemicarbazone groups oriented in opposite directions like a two-blade propeller, thereby forming a peculiar chiral helicity. A water molecule has been found, in the center of the two-blade propeller, strongly coordinated through multiple H-bonds. A considerable antibacterial activity against various tested microorganisms (gram-positive bacteria: *S. aureus* and *B. subtilis*; gram-negative bacteria: *E. coli* and *P. aeruginosa*; and fungi: *C. albicans* and *C. glabrata*) was observed for the ligand and for all its metal derivatives (Co^2+^, Ni^2+^, Cu^2+^, and Zn^2+^). Interesting antifungal activities against yeast (*C. albicans*) were also observed for the ligand and for its Co^2+^ derivative. All compounds showed an increasing cytotoxicity against the tested cancerous cells (Saos-2 and MG-63) with increasing concentration, with the exclusion of Zn^2+^ derivative against Saos-2 cells. Interestingly, for the Saos-2 cell line, the anticancer activity of ligand L was higher than its metal derivatives at all concentrations. Microscopic analysis of DAPI-stained cells showed changes in morphology, with deformation and fragmentation of the nuclei. Finally, the hemo-compatibility study demonstrated that all of the compounds have a very slight lysis activity, which would make them suitable candidates for further in vivo investigations.

## Figures and Tables

**Table 1:** Summary of the main short interactions found in the structure of **L**.

D •••A ^a^	d (D•••A) (Å)
**Intramolecular H-Bonds on Calix[4]arene Lower Rim**	
O(1B)•••O(1A)	2.871
O(1D)•••O(1C)	2.922
**Intramolecular H-Bonds on Thiosemicarbazone Substituents**	
O(2A)•••N(3A)	2.710
O(2C)•••N(3C)	2.742
**Interactions between Water Molecule and Thiosemicarbazone Substituents ^b^**	
N(1A)•••O(1W)	2.978
N(1C)•••O(1W)	3.185
O(2A)•••O(1W)	2.850 (phenol hydroxy)
O(2C)•••O(1W)	3.051 (phenol hydroxy)
**Interactions between Water Molecule and Calix[4]arene Lower Rim Oxygen Atoms ^b^**	
O(1D)•••O(1W)	2.872 (hydroxy)
O(1B)•••O(1W)	2.986 (hydroxy)
O(1C)•••O(1W)	3.094 (alkoxy)
O(1A)•••O(1W)	3.258 (alkoxy)

^a^ The atom labels in parenthesis are those used in the deposited structure. ^b^ For clarity, the type of non-water oxygen atom involved is identified in parenthesis.

**Table 2:** Antibacterial activity of synthesized compounds against different microorganisms in the micro-broth dilution method. The antibacterial activity is expressed as the MIC/MBC (μg/mL).

*Microorganism*	*S. aureus*	*B. subtilis*	*E. coli*	*P. aeruginosa*	*C. albicans*	*C. glabrata*
Ligand **L**	-	31.25	31.25/31.25	31.25/-	31.25/125	-
Complex **Co**	-	31.25/1000	31.25/31.25	31.25/250	62.5/-	-
Complex **Ni**	-	31.25	31.25/31.25	31.25/2000	-	-
Complex **Cu**	31.25	31.25	31.25/31.25	31.25/500	-	-
Complex **Zn**	-	31.25	31.25/31.25	31.25/31.25	-	-
Gentamicin *	0.12/0.25	2/2	0.5/0.5	2/2	-	-
Nystatin *	-	-	-	-	1.25	0.625

*Staphylococcus aureus* (ATCC^®^ 29213™), *Bacillus subtilis* (ATCC^®^ 6633™), *Escherichia coli* (ATCC^®^ 25922™), *Pseudomonas aeruginosa* (ATCC^®^ 27853™), *Candida albicans* (ATCC^®^ 10231™), and *Candida glabrata* (ATCC^®^ 2001™). * Gentamicin and nystatin used as a standard control for bacteria and fungi, respectively.

**Table 3:** In vitro cytotoxic activity IC_50_ (μg/mL) of compounds against bone cancer cell lines.

Compound	Saos-2	MG-63
Ligand **L**	<25	>200
Complex **Co**	80	195
Complex **Ni**	62	173
Complex **Cu**	43	140
Complex **Zn**	>200	>200
MTX	7.9	14.5
DOX	2.1	10.1

## Supplementary Materials

The Supplementary Materials are available online.
