# Supplementary material for: Synthesis, Crystal Structure, and Biological Activity of a Multidentate Calix[4]arene Ligand Doubly Functionalized by 2-Hydroxybenzeledene-Thiosemicarbazone
Source: Molecules. 2020 Jan 16;25(2):370. doi: 10.3390/molecules25020370 (PMC7024204; doi:10.3390/molecules25020370)
Supplement: Supplementary file 1 [file molecules-25-00370-s001.pdf]

Supporting Information

Thiosemicarbazone appended calix[4]arene in 1, 3-distal configuration: synthesis, crystal structure, transition metal complexes with insights into antimicrobial and anticancer activity

Ehsan Bahojb Noruzi,[a] Behrouz Shaabani,*[a] Silvano Geremia,[b] Neal Hickey,[b] Patrizia Nitti,[b] Hossein Samadi Kafil[c]

[a] Faculty of Chemistry, Department of Inorganic Chemistry, University of Tabriz, Tabriz, Iran

[b] Department of Chemical and Pharmaceutical Sciences, University of Trieste, Trieste, Italy

[c] Drug Applied Research Center, Tabriz University of Medical Sciences, Tabriz, Iran.

FT-IR Spectra of L and L-Co, L-Ni, L-Cu, L-Zn derivatives

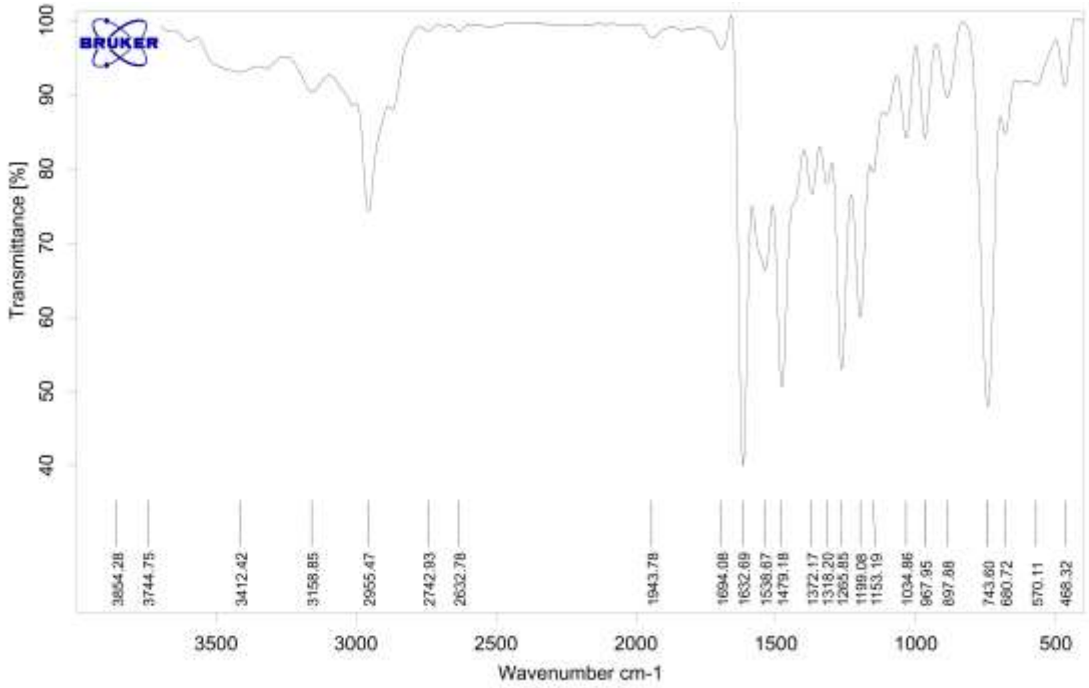

Figure S1: FT-IR spectrum of **L** (KBr pellet).

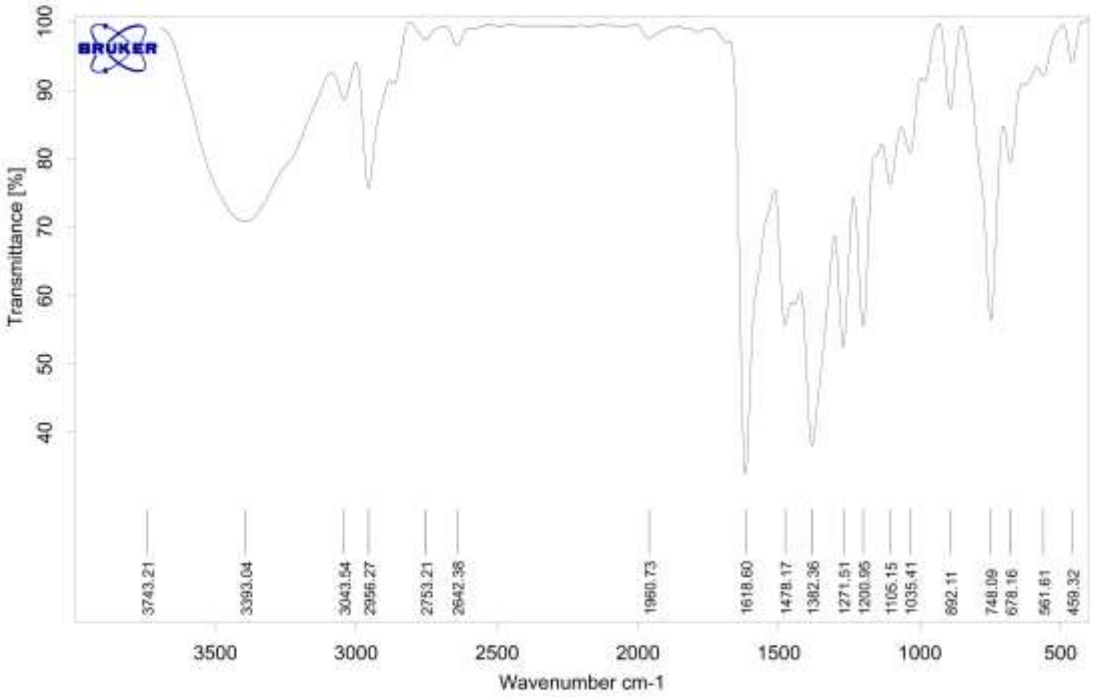

Figure S2: FT-IR spectrum of **L-Co** derivative (KBr pellet).

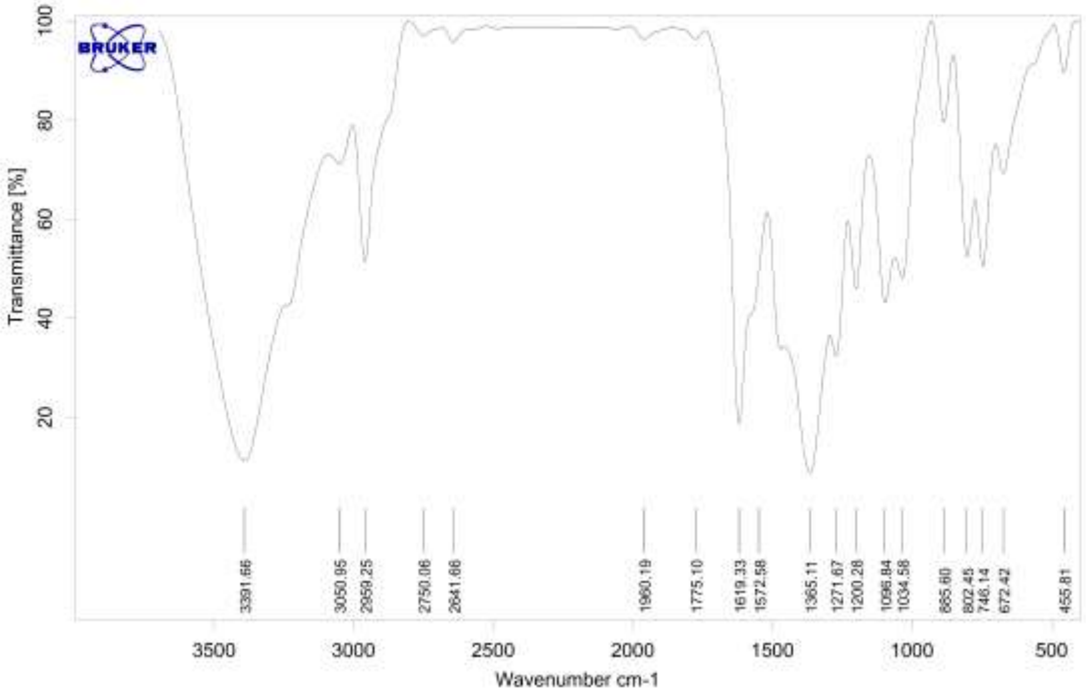

Figure S3: FT-IR spectrum of **L-Ni** derivative (KBr pellet).

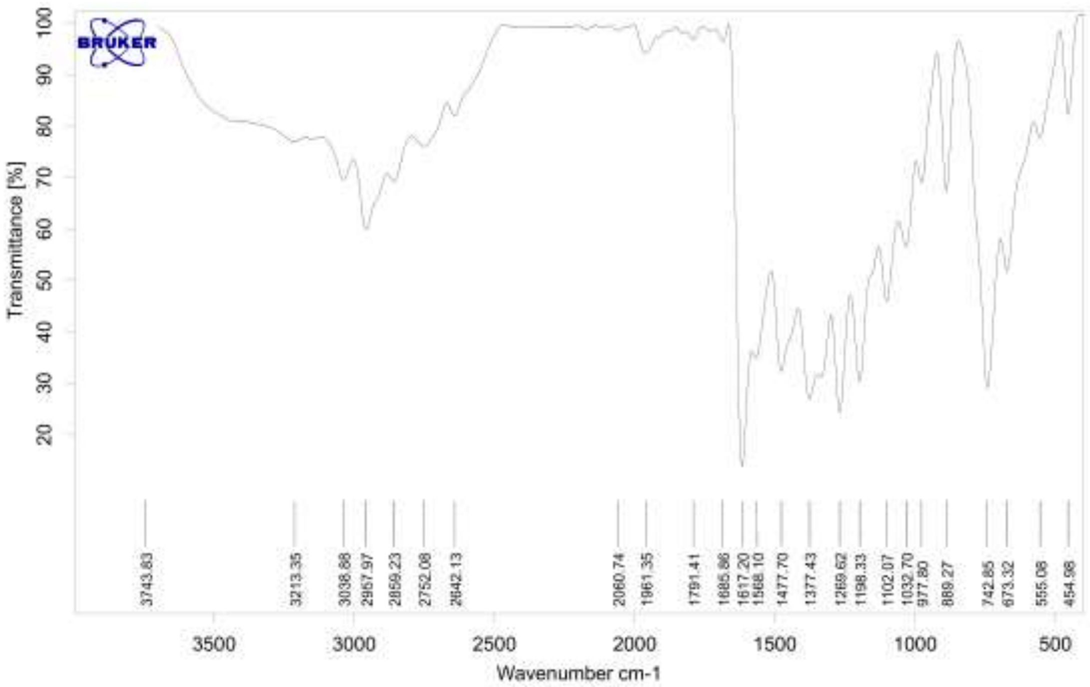

Figure S4: FT-IR spectrum of **L-Cu** derivative (KBr pellet).

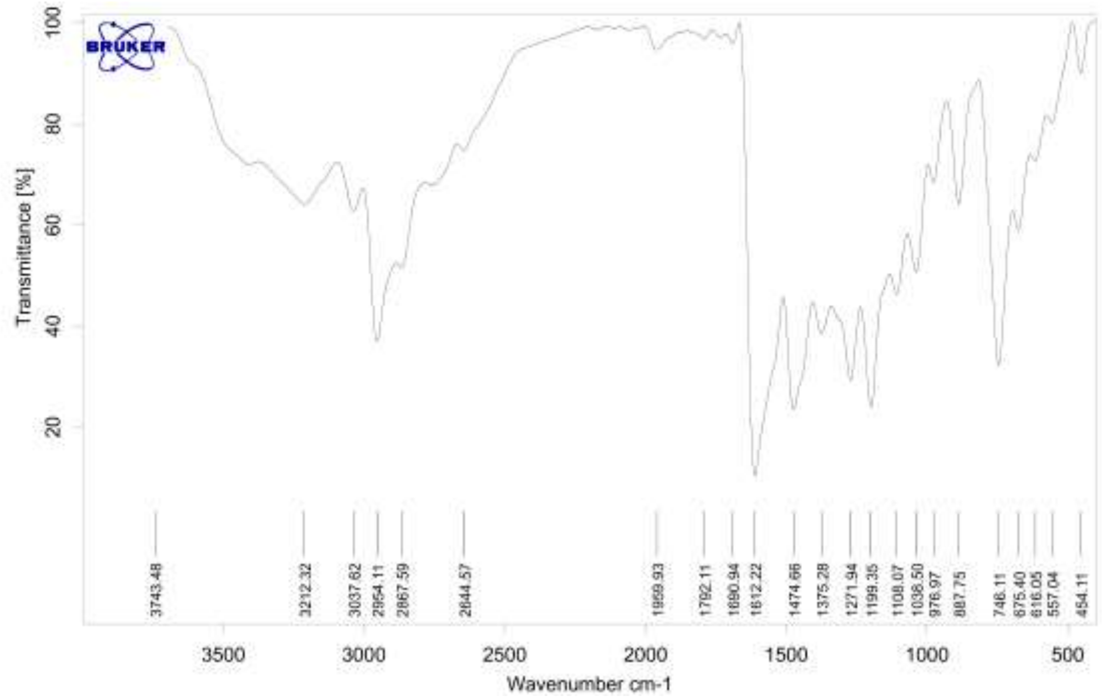

Figure S5: FT-IR spectrum of **L-Zn** derivative (KBr pellet).

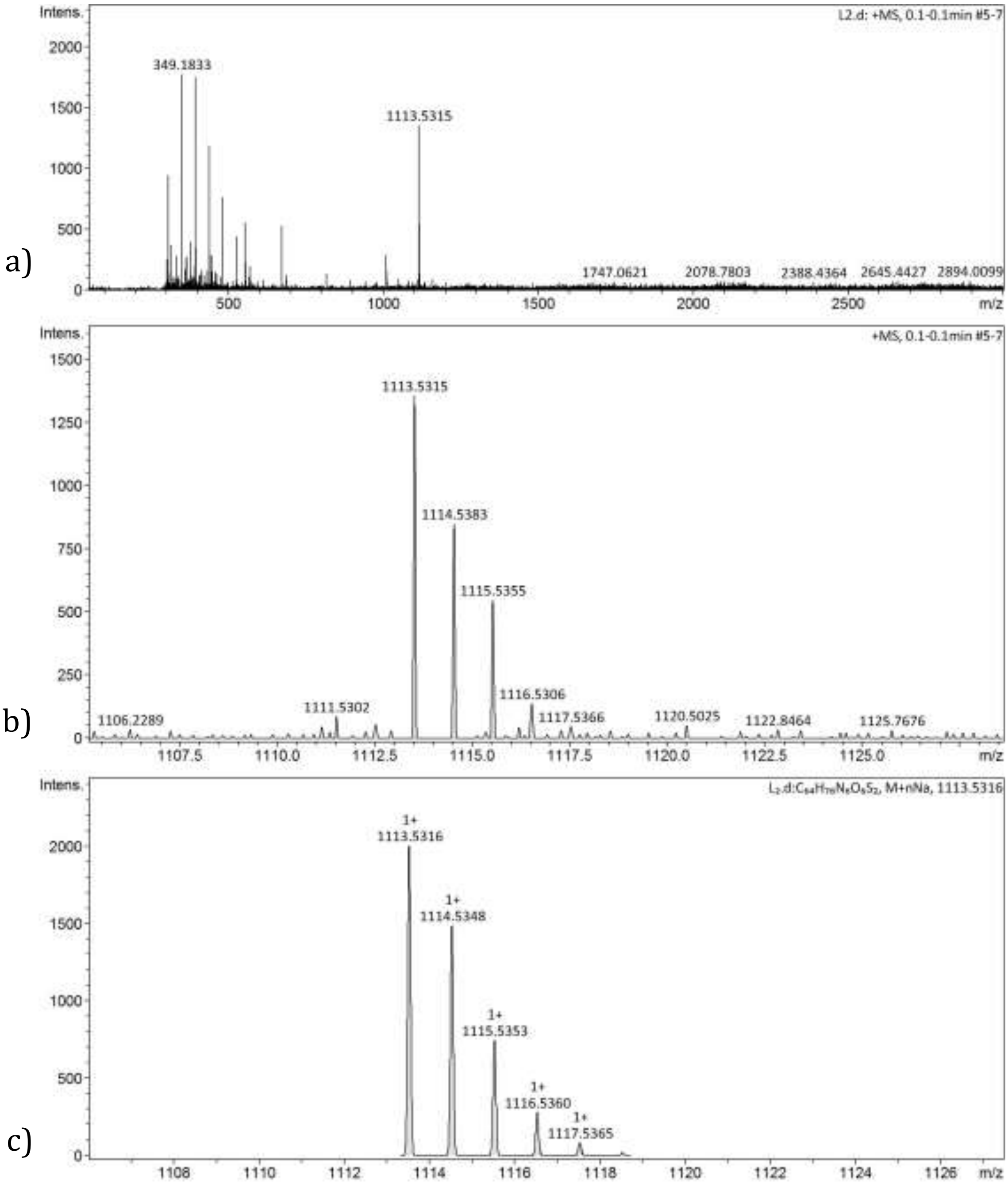

Figure S6: a) Positive-ion ESI High Resolution Mass Spectra (HRMS) of **L**. Observed (b) and calculated (c) isotopic distributions for LNa^+, $[C_{64}H_{78}N_6O_6S_2Na]^+$.

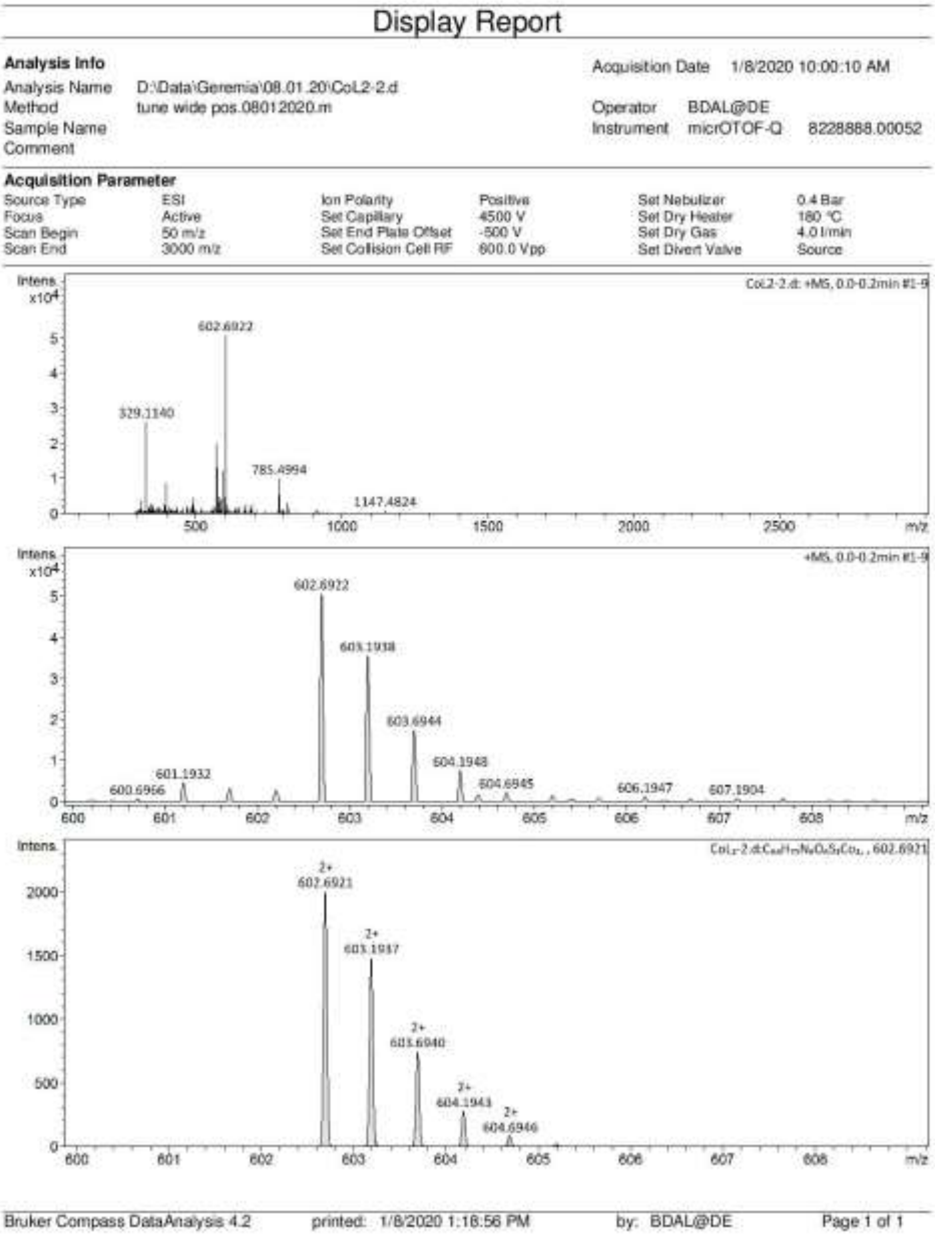

Figure S7: Positive-ion ESI High Resolution Mass Spectra (HRMS) of Co^{2+} derivative of L. Observed (middle) and calculated (bottom) isotopic distributions for $LCo(II)Co(III)^{2+}$, $[C_{64}H_{75}N_6O_6S_2Co_2]^{2+}$.

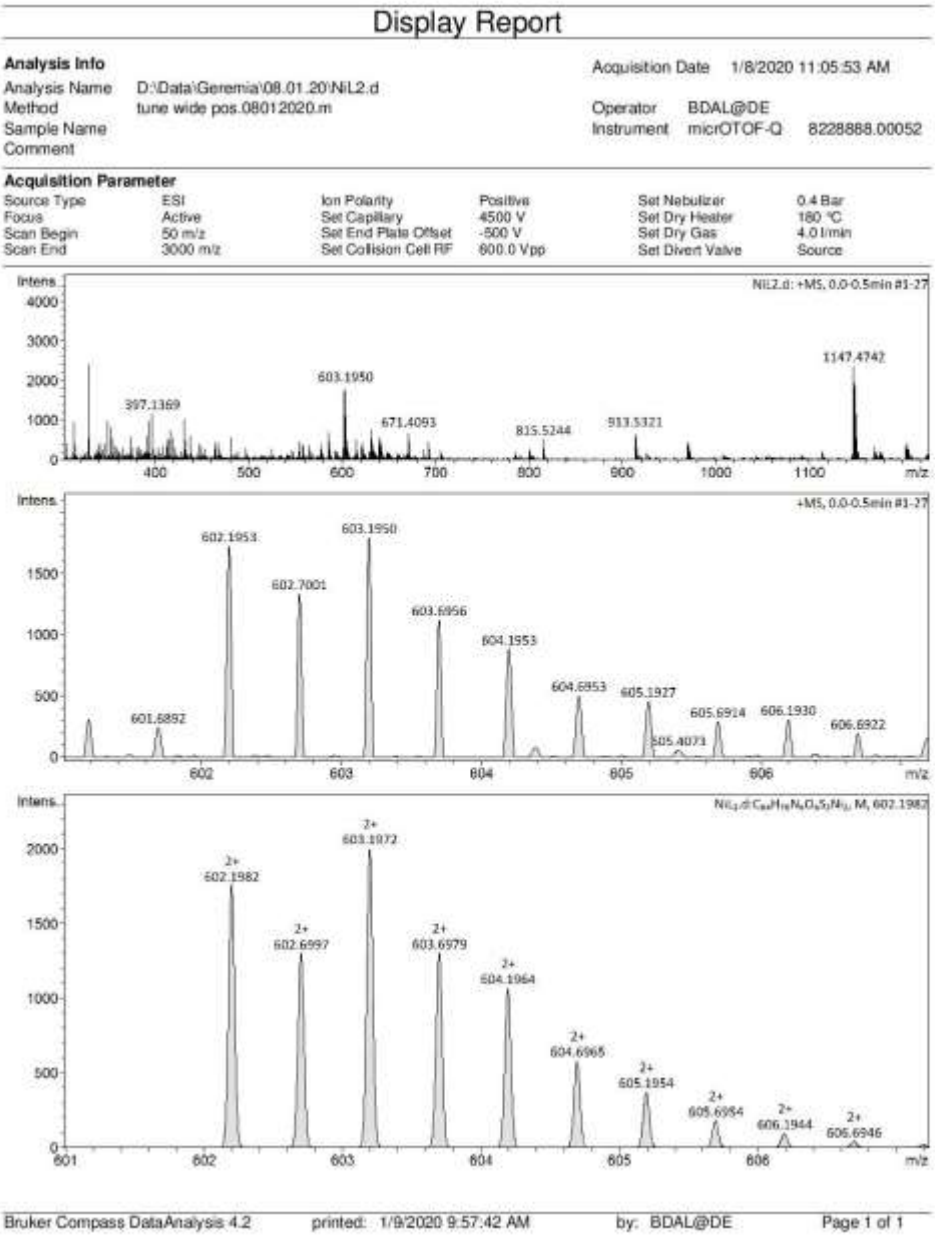

Figure S8: a) Positive-ion ESI High Resolution Mass Spectra (HRMS) of Ni^{2+} derivative of L. Observed (middle) and calculated (bottom) isotopic distributions for $LNi(II)_2^{2+}$, $[C_{64}H_{76}N_6O_6S_2Ni_2]^{2+}$.

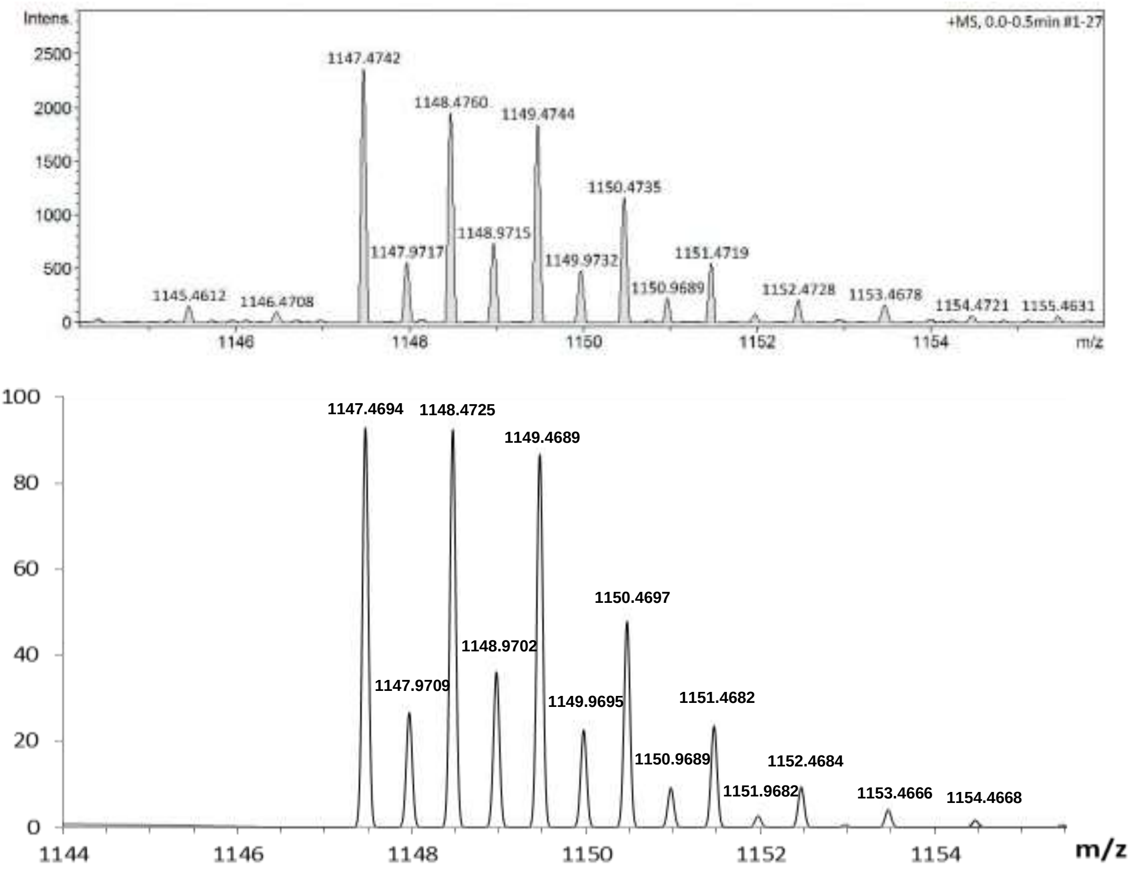

Figure S9: Observed (top) and calculated (bottom) isotopic distributions for a mixture containing 0.75 LNi(II)$^{+}$, $[C_{64}H_{77}N_6O_6S_2Ni]^{+}$ and 0.25 $L_2Ni(II)_2^{2+}$, $[C_{128}H_{154}N_{12}O_{12}S_4Ni_2]^{2+}$. Masses were calculated by enviPat Web 2.2 (http://www.envipat.eawag.ch/index.php).

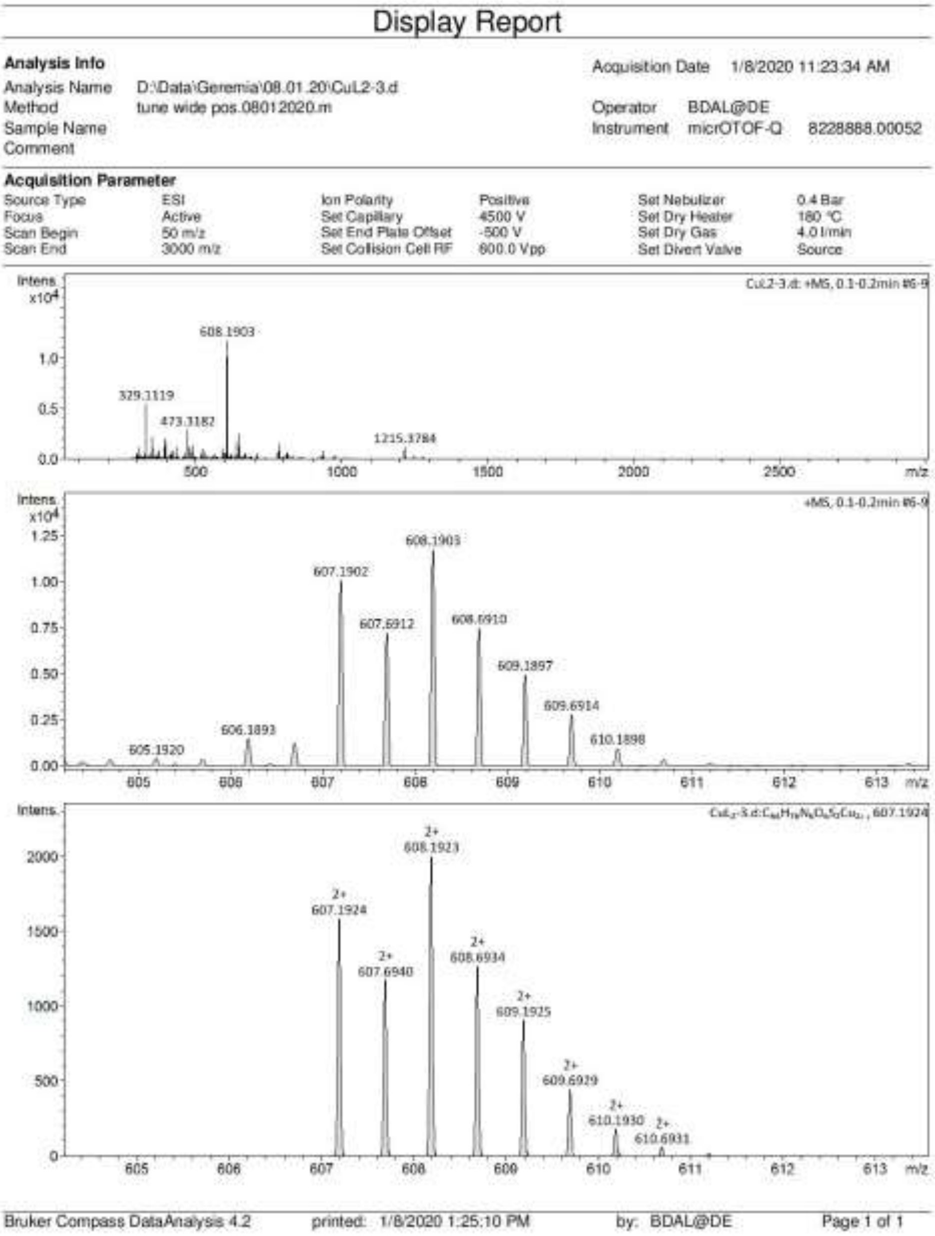

Figure S10: Positive-ion ESI High Resolution Mass Spectra (HRMS) of Cu^{2+} derivative of L. Observed (middle) and calculated (bottom) isotopic distributions for $LCu(II)_2^{2+}$, $[C_{64}H_{76}N_6O_6S_2Cu_2]^{2+}$.

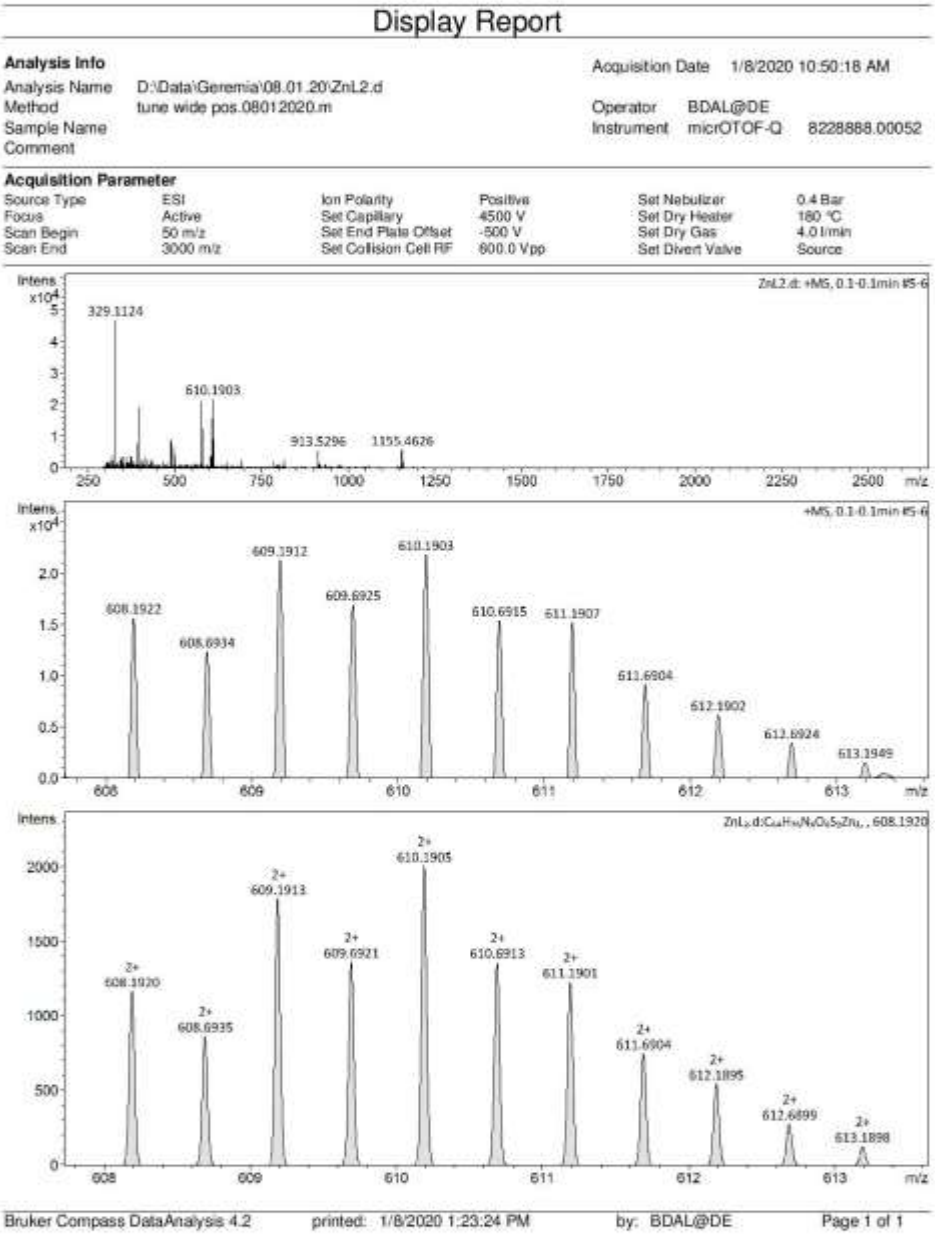

Figure S11: Positive-ion ESI High Resolution Mass Spectra (HRMS) of Zn^{2+} derivative of L. Observed (middle) and calculated (bottom) isotopic distributions for $LZn(II)_2^{2+}$, $[C_{64}H_{76}N_6O_6S_2Zn_2]^{2+}$.

Table S1. IR spectral data (cm^{-1}) of the ligand and its corresponding metal derivatives in KBr pellets.

Vibrational mode	Frequency in (cm^{-1})				
	**L**	**LCo^{2+}**	**LNi^{2+}**	**LCu^{2+}**	**LZn^{2+}**
**C=N**	1633	1618	1619	1617	1612
**N-C=S**	1539	shoulder	1572	1568	shoulder
**C=S**	898	892	885	889	887

Table S2. Crystal data and structure refinement for compound L.

	**Ligand L**
Empirical formula	($C_{64}H_{78}N_6O_6S_2$), H_2O
Formula weight	1109.46
Temperature (K)	100(2)
Wavelength (Å)	0.7
Crystal system	Triclinic
Space group	*P* -1
Unit cell dimensions (Å, °)	a = 12.690(6), α = 104.44(4)
	b = 15.344(18), β = 98.01(4)
	c = 19.15(3), γ = 104.016(10)
Volume ($Å^3$)	3423(6)
Z	2
ρ_{calcd} (g/cm^3)	1.076
μ (mm^{-1})	0.122
F(000)	1188
Reflections collected	23853
Independent reflections	6918 [R(int) = 0.1328]
Data / restraints / parameters	6918 / 3 / 734
GooF	0.972
Final *R* indices [I>2σ(I)]	R_1 = 0.0683, wR_2 = 0.1677
R indices (all data)	R_1 = 0.1359, wR_2 = 0.2108
CCDC code	1944727

Table S3: Elemental analysis of the metal derivatives of **L**

<table>
<tr><td colspan="4">L</td></tr>
<tr><td colspan="4">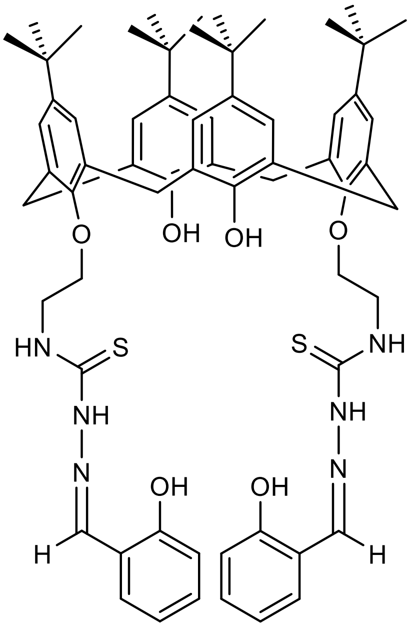
</td></tr>
<tr><th>Metal salt</th><th>Proposed structure</th><th colspan="2">Elemental analysis</th></tr>
<tr><td rowspan="2">$Co(NO_3)_2.6H_2O$</td><td rowspan="2">$[CoL](NO_3)_2.2H_2O$
$C_{64}H_{82}N_8O_{14}S_2Co$</td><td>C, 58.66; H, 6.31; N, 8.55</td><td>Calculated</td></tr>
<tr><td>C, 58.76; H, 5.97; N, 8.43</td><td>Found</td></tr>
<tr><td rowspan="2">$Ni(NO_3)_2.6H_2O$</td><td rowspan="2">$[NiL](NO_3)_2$
$C_{64}H_{78}N_8O_{12}S_2Ni$</td><td>C, 60.33; H, 6.17; N, 8.79</td><td>Calculated</td></tr>
<tr><td>C, 60.32; H, 5.95; N, 8.99</td><td>Found</td></tr>
<tr><td rowspan="2">$Cu(NO_3)_2.2H_2O$</td><td rowspan="2">$[CuL](NO_3)_2$
$C_{64}H_{78}N_8O_{12}S_2Cu$</td><td>C, 60.10; H, 6.15; N, 8.76</td><td>Calculated</td></tr>
<tr><td>C, 59.72; H, 5.98; N, 8.66</td><td>Found</td></tr>
<tr><td rowspan="2">$Zn(NO_3)_2.4H_2O$</td><td rowspan="2">$[ZnL](NO_3)_2$
$C_{64}H_{78}N_8O_{12}S_2Zn$</td><td>C, 60.01; H, 6.14; N, 8.75</td><td>Calculated</td></tr>
<tr><td>C, 60.23; H, 6.01; N, 8.64</td><td>Found</td></tr>
</table>
